# Impact of exercise sequence in concurrent training on insulin resistance, glycemic control, and blood pressure in Type 2 diabetes

**DOI:** 10.1371/journal.pone.0340587

**Published:** 2026-01-07

**Authors:** Friew Amare, Alemmebrat Kiflu, Aschenaki Taddese

**Affiliations:** Department of Sport Science and Physical Education, College of Education and Language Studies, Addis Ababa University, Addis Ababa, Ethiopia; Federal University of Pernambuco: Universidade Federal de Pernambuco, BRAZIL

## Abstract

This study compared the effects of aerobic followed by resistance (CART) and resistance followed by aerobic (CRAT) training on insulin sensitivity, glycemic control, and blood pressure against a non-exercising control group (COT). 39 patients with type 2 diabetes were randomly assigned to CART (n = 13), CRAT (n = 13), or COT (n = 13) for a 12-week supervised program. Homeostatic Model Assessment of Insulin Resistance (HOMA-IR) improved significantly (F = 24.460, p = 0.001, η² = 0.62), with CRAT showing a modestly greater reduction than CART (MD = 0.303, p = 0.022), while both training sequences produced greater improvements compared to COT (CART: MD = −0.493, p = 0.001; CRAT: MD = −0.796, p = 0.001). Fasting Blood Sugar (FBS) decreased significantly (F = 16.206, p = 0.001, η² = 0.519) in both CART (MD = −10.747, p = 0.001) and CRAT (MD = −11.459, p = 0.001) relative to COT, though no difference emerged between the two exercise sequences (MD = 0.712, NS). Systolic Blood Pressure (SBP) also declined in CART (MD = −4.896, p = 0.005) and CRAT (MD = −5.681, p = 0.001) compared with COT (F = 8.968, p = 0.001, η² = 0.374), again without a significant sequence effect (MD = 0.785, NS). No significant between-group changes were observed in Diastolic Blood Pressure (DBP). Overall, concurrent training effectively reduced insulin resistance, improved glycemic control, and lowered SBP in patients with type 2 diabetes. While CRAT offered a modest additional benefit for HOMA-IR, the findings suggest that concurrent training, regardless of sequence, provides meaningful clinical benefits for managing type 2 diabetes.

**Trial registrationPan African Clinical Trials Registry (PACTR202509591505325)** pactr.samrc.ac.za/TrialDisplay.aspx?TrialID=37070

## Introduction

Type II diabetes mellitus (T2DM) is a chronic metabolic disorder characterized primarily by insulin resistance, impaired glucose regulation, and an elevated risk of cardiovascular complications [1,2]. Insulin resistance (IR), the diminished effectiveness of insulin in promoting glucose uptake by peripheral tissues [3], plays a pivotal role in the development and progression of T2DM [4]. This defect leads to sustained high blood glucose levels and triggers a series of metabolic disruptions that contribute to the onset of diabetes-related complications, including cardiovascular disease [5], making IR a critical target for therapeutic interventions. Beyond glycemic impairment, individuals with T2DM frequently exhibit altered hemodynamic parameters such as elevated systolic and diastolic blood pressure (SBP, DBP), which further exacerbate cardiovascular risk and contribute to disease progression [6,7].

Exercise training, particularly concurrent training that integrates aerobic and resistance modalities, has proven to be an effective nonpharmacological strategy for enhancing insulin sensitivity, controlling blood glucose, and improving blood pressure regulation [8,9]. Recent evidence suggests that the sequence in which aerobic and resistance exercises are performed can trigger different physiological adaptations [10,11], potentially influencing the degree of improvement in insulin resistance, blood glucose regulation, and hemodynamic parameters. Mechanistic pathways implicated include variations in hormonal responses [12], muscle fiber recruitment, body composition and metabolic demand contingent on exercise order [13,14].

Despite these insights, there remains a paucity of research directly comparing the effects of different exercise sequences within concurrent training on insulin resistance, fasting glucose (FG), SBP, and DBP in adults with T2DM. Most existing studies have focused on isolated exercise modalities or combined effects without systematically evaluating the role of exercise order in optimizing metabolic and cardiovascular adaptations [8,9,15]. Furthermore, potential confounding factors such as medication regimens, dietary intake, and baseline fitness levels have often been inadequately controlled, limiting the generalizability and clinical application of findings.

Addressing this gap is critical because insulin resistance and hemodynamic abnormalities are interlinked determinants of diabetes-related morbidity and mortality, and optimizing exercise protocols to target these parameters specifically could substantially improve patient outcomes. Therefore, this study aims to investigate how the sequence of aerobic and resistance training within a concurrent exercise program affects insulin resistance, fasting glucose, and blood pressure markers in adults with type II diabetes. The results are anticipated to inform evidence-based guidelines for structuring concurrent training regimens to maximize metabolic and cardiovascular health benefits in this vulnerable population.

## Methods and materials

The study employed a 12-week parallel-group randomized controlled trial design to investigate the effects of exercise sequence during concurrent training. Adults with type II diabetes were randomly assigned in a 1:1:1 ratio to one of three groups: CART (Concurrent Aerobic-Resistance Training), CRAT (Concurrent Resistance-Aerobic Training), or a wait-list control group (COT). The random allocation sequence was generated by an independent statistician not involved in participant recruitment or outcome assessment. A computer-generated randomization sequence was used to assign participants. The random allocation sequence was implemented using sequentially numbered, opaque, sealed envelopes (SNOSE) prepared by an independent statistician. Each envelope contained the group assignment and was stored securely. Randomization was stratified by sex and age (41–60 years), and outcome assessors were blinded to group allocation. The intervention was held three times a week (on Tuesdays, Thursdays, and Sundays) at 5:00 PM. While the exercise type and duration were the same for both treatment groups. The primary result was arterial stiffness, while the secondary outcome was lipid profile, along with other cardiometabolic indicators tested before and after. Participant recruitment was conducted from June 1 to June 10, 2025, and the intervention period lasted from June 17 to September 8, 2025. Follow-up assessments were completed immediately after the intervention period.

The Declaration of Helsinki’s guiding principles were followed in the conduct of this investigation. The Institutional Review Board of Debre Markos University’s Sport Sciences Academy approved the protocol (Reference No. SpScAc.IRC/03/2025). Before enrollment, each subject gave written informed consent. Due to administrative delays and a lack of knowledge about registration procedures at the time, the trial registration (Registration No: PACTR202509591505325) was completed during the intervention period. Nonetheless, no significant protocol changes occurred after recruitment, and the registered protocol accurately reflects the methods used.

Patients were not directly involved in the design, conduct, reporting, or dissemination plans of this trial. However, during the preparatory phase, feedback was obtained from diabetes clinic staff and exercise specialists to ensure the intervention protocol was safe, feasible, and culturally appropriate for Type II diabetic patients. Participants were informed about the study purpose and procedures before giving written informed consent, and their perspectives on exercise feasibility were noted during follow-up.

**Fig 1:**
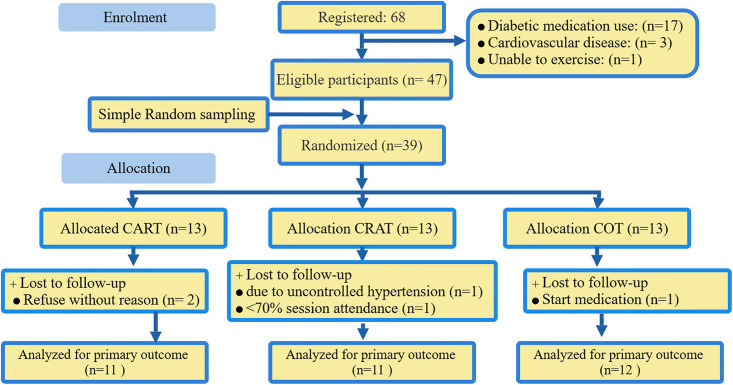
Participant flow diagram (According to CONSORT 2025 Guidelines) [16].

As displayed in (Fig 1), 68 adults with physician-confirmed type 2 diabetes mellitus (T2DM) were recruited from the outpatient department of Debre Markos Referral Hospital and the surrounding community. Recruitment strategies included hospital records, local radio announcements, posters, and brochures. 47 eligible individuals were screened using predefined inclusion and exclusion criteria, and written informed consent was obtained from all participants. Ethical principles such as confidentiality, voluntary participation, and medical clearance for exercise participation were strictly observed in accordance with ins0titutional guidelines.

Adults between the ages of 41 and 60 who had been diagnosed with type 2 diabetes mellitus (T2DM), were physically inactive, were managing their condition with dietary and/or lifestyle changes, and had received medical clearance to take part in physical activity using the Physical Activity Readiness Questionnaire (PAR-Q) were eligible participants. Exclusion criteria included diabetes-related complications (such as neuropathy, nephropathy, or retinopathy), uncontrolled hypertension (>220/105 mmHg), hyperglycemia (>250 mg/dl), diagnosed cardiovascular disease, or any musculoskeletal or chronic conditions that would limit safe participation in exercise. Additionally, participants who attended less than 70% of exercise sessions were excluded from the final analysis [17].

The trial was conducted at the Debre Markos University Sport Science Academy Exercise Laboratory and the nearby community fitness facility equipped with aerobic and resistance training machines. Sites were eligible if they had adequate space, safety measures, and certified exercise equipment to ensure consistent delivery of the concurrent training protocols.

Exercise sessions were supervised by qualified sport science professionals holding at least a bachelor degree in exercise science or related fields, with prior experience in supervising exercise programs for patients with chronic metabolic diseases. Intervention providers were required to complete a study-specific orientation on the exercise protocol, safety monitoring, and emergency procedures before initiating participant training.

Clinical assessments and biochemical sampling were performed by trained nurses and laboratory technicians under the supervision of the principal investigator.

Sample size was calculated using G*Power software (version 3.1.9.7), based on a previously reported effect size of 0.41 for changes in total cholesterol. With an alpha level of 0.05 and a statistical power of 80%, the minimum required sample was 36 participants. Allowing for a 10% dropout rate, the final target was set at 39 participants, with 13 allocated to each intervention group. Eligible individuals were randomly selected from the diabetic patient registry using simple random sampling to minimize selection bias and ensure equal representation.

### Measurement of study variables

The primary outcome of this study was insulin resistance (IR), evaluated at baseline and after the 12-week intervention. Secondary outcomes included fasting glucose (FG) and hemodynamic indices, such as systolic blood pressure (SBP) and diastolic blood pressure (DBP), measured at the same time points. The independent variable was the sequence of exercise within the concurrent training program, specifically comparing aerobic-resistance with resistance-aerobic. To account for its possible impact on metabolic and cardiovascular reactions, average daily energy intake was also included as a covariate in the statistical analysis.

### Measurement of fasting glucose and insulin resistance

As indicated in Equation 1, IR was assessed using the Homeostasis Model Assessment of Insulin Resistance (HOMA-IR), a validated index that combines fasting glucose and fasting insulin concentrations to estimate insulin sensitivity [18]. Blood samples for fasting insulin and glucose were collected from the antecubital vein by a trained medical laboratory technician, 48 hours after the most recent training session, to minimize acute exercise effects. Participants were instructed to fast for at least 12 hours and to refrain from alcohol, coffee, and high-fat meals before the test.

A single blood draw was used to obtain serum for both fasting insulin and fasting glucose analysis. Fasting insulin was measured using the Enzyme-Linked Immunosorbent Assay (ELISA) method type China. [19] while fasting glucose was determined via the enzymatic colorimetric method on an Alpha X autoanalyzer (Hitachi, Tokyo, Japan). Since fasting insulin assays were not available at Debre Markos Referral Hospital, serum samples were transported under cold chain conditions to Wudassie Diagnostic Center in Addis Ababa for analysis.

The HOMA-IR index was calculated using the standard formula Eqe (1) [20].


$$HOMA-IR=\frac{\text{fasting}\;\text{serum}\;\text{glucose}\;(\text{mg}.{\text{dL}}^{-\text{1}})*\text{fasting}\;\text{serum}\;\text{insulin}(\mu \text{U}.{mL}^{-\text{1}})}{\text{40}\text{5}}$$ (1)

*Equation 1: Homeostasis model of insulin resistance equation*

### Blood pressure measurements

Blood pressure was measured using an automated SphygmoCor XCEL device (AtCor Medical, Sydney) [21], was adapted from Mengistu, Lake [9]. The cuff size was chosen based on each participant’s arm circumference. Participants avoided caffeine, alcohol, and exercise for at least 30 minutes before the test and rested lying down for 5 minutes. The cuff was placed on the left upper arm, aligned with the brachial artery, and positioned 2–3 cm above the elbow crease at heart level. Three readings were taken consecutively, and the average of the last two was used. Participants stayed still and quiet during measurements.

### Covariate variable

While nutrition was not the study’s primary focus, participants’ average daily calorie intake was monitored because it might impact the results. Dietary data were collected using a validated multiple-pass 24-hour recall method, which includes five steps: initial list, forgotten items, timing and context, detailed probing, and final review. This assessment was done on three separate days, two weekdays and one weekend day. Energy and nutrient content were estimated using the Ethiopian Food Composition Table and analyzed with NutriSurvey200 software. The mean energy intake over the three days was used for analysis.

### Exercise training protocol

The concurrent training program consisted of 36 individually supervised sessions conducted three times weekly on alternating days (Tuesday, Thursday, and Sunday) over 12 weeks (APPENDIX VII). Each 70-minute session included a 5-minute warm-up, 60 minutes of main exercise (30 minutes aerobic and 30 minutes resistance), and a 5-minute cool-down. The program followed the American College of Sports Medicine (ACSM, 2020) guidelines for individuals with type II diabetes.

Before the intervention, participants underwent a one-week familiarization phase featuring low-intensity aerobic exercises on a treadmill (walking, jogging) and low-load resistance exercises performed for 10–15 repetitions without fatigue, to reduce bias and ensure safety.

The resistance training (RT) targeted major muscle groups in accordance with American Diabetes Association recommendations (Sigal et al., 2006). Exercises included abdominal curls, standing plantar flexion, bodyweight squats, machine leg press, neutral rowing, bicep curls, triceps pulley, and machine bench press using multi-gym equipment (Cybex International, Medway, MA). Resistance ranged from moderate to high intensity, and circuit-style RT was performed with 15–20 second rests between sets, involving 1–3 sets of 10–15 repetitions to near fatigue, with 30–90 second breaks between exercises. The load was progressively increased to maintain training intensity.

Aerobic training utilized a Cybex treadmill (Cybex Corporation, NY) at moderate (40–59% HRR) to vigorous (60–89% HRR) intensity [22]. Target heart rates were managed using the heart rate reserve (HRR) method based on the Karvonen formula. Heart rates were continuously monitored during exercise using Polar H7 heart rate monitors (Polar Electro, Finland) to ensure participants maintained the prescribed intensity (Hernández-Vicente et al., 2021).

Two groups performed concurrent training differing only in exercise order: concurrent resistance-aerobic training (CRAT) and concurrent aerobic-resistance training (CART). A 5-minute rest period separated the aerobic and resistance components during each session.

To ensure the detection of any adverse events, heart rate, blood pressure, and blood glucose levels will be monitored before and after each exercise session, particularly if a medical professional observes unusual or concerning patterns. It would not be possible to incorporate a placebo therapy that mimicked exercise in this investigation (Arora et al., 2023). Rather, a control group will be created in which individuals continue with their regular care regimen while abstaining from any regular exercise program. This makes it possible to compare the impact of the training program with the individuals’ current diabetes control techniques.

### Statical analysis

Data were analyzed using SPSS version 27 (SPSS Inc., Chicago, IL). For each group, concurrent aerobic-resistance training (CART), concurrent resistance-aerobic training (CRAT), and control group (COG), means, standard deviations (SDs), and normality tests were calculated at baseline.

A repeated measures ANCOVA (RM-ANCOVA) with Bonferroni correction for multiple comparisons was performed, including average daily energy intake as a covariate. The primary outcome analyzed was the Homeostasis model of insulin resistance (HOMA-IR). Secondary outcomes included fasting blood sugar (FBS), systolic blood pressure (SBP), and diastolic blood pressure (DBP). Changes over time within participants and differences between groups were assessed, along with interactions between time and group effects. All tests were two-tailed, with statistical significance set at p ≤ 0.05.

### Interim analyses and stopping guidelines

During the trial, no interim analyses were planned or carried out. All analyses were carried out immediately after data collection was finished because the study was planned with a modest sample size and a relatively short intervention period (12 weeks).

There was no official data monitoring committee or stopping instructions for the trial. However, the lead investigator and supervising exercise specialists kept a close eye on participant safety. The afflicted participant’s training would have been stopped, and the necessary medical attention would have been given if there had been any significant adverse events (such as severe hypoglycemia, a cardiovascular event, or an injury). If there had been several major incidents, the trial would have been suspended while it was reviewed ethically.

### Changes to trial after commencement

No major changes were made to the trial design, eligibility criteria, interventions, or outcome measures after the study commenced. However, minor adjustments were made to the training schedule to accommodate participants’ availability and ensure adherence during the 2 weeks of the orthodox church fasting weeks. These adjustments did not affect the exercise intensity, sequence, or total training volume.

## Results

Table 1 presents a summary of the characteristics of subjects at both baseline and follow-up. A total of 39 participants were randomized (CART = 13, CRAT = 13, COT = 13). Due to attrition, 11 participants from CART, 11 from CRAT, and 12 from COT were included in the final analysis. Table 1 presents the baseline and follow-up characteristics of the study groups. The groups were similar in terms of age (CART: 48.7 ± 2.1 years; CRAT: 49.7 ± 3.0 years; COT: 49.0 ± 2.9 years), sex distribution (72–83% male), BMI (approximately 27.5–27.7 kg/m²), and daily caloric intake (2381–2515 kcal). At the follow-up, HOMA-IR decreased in the CART group from 3.93 to 3.36 and in the CRAT group from 4.07 to 2.61, while it stayed stable in COT at around 4.1. Fasting blood sugar levels also fell in CART from 132.2 to 118.0 mg/dL and in CRAT from 134.7 to 113.9 mg/dL, but demonstrated no change in COT (134.2 to 135.4 mg/dL). Likewise, systolic and diastolic blood pressure readings were lowered in CART from 130.7 to 124.5 mmHg and from 85.7 to 82.5 mmHg, and in CRAT from 132.9 to 120.8 mmHg and from 87.3 to 80.7 mmHg, while minor increases were noted in COT. Baseline comparisons of key biomarkers, including HOMA-IR, fasting blood sugar (FBS), and blood pressure (SBP and DBP), showed no statistically significant differences between groups (p > 0.05), indicating that the groups were well-matched at the start of the intervention.

**Table 1:** Baseline and follow-up demographic and clinical characteristics of participants by group.

VARIABLES	CART		CRAT		COT	
	Baseline	Follow-up	Baseline	Follow-up	Baseline	Follow-up
Age (years)	48.73 ± 2.054		49.732.970		49.002.923	
Sex (male), n (%)	9 (81.8%)		8 (72.7%)		10 (83.3%)	
BMI (kg/m^2^)	27.55 ± .99		27.52 ± .67		27.69 ± .88	
ADEi (kcal)	2381.15 ± 147.52		2389.1845116.85683		2515.29 ± 72.96	
HOMA-IR	3.93 ± .39	3.36 ± .16	4.07 ± .27	2.61 ± .20	4.09 ± .29	4.14 ± .29
FBS (mg/ dL)	132.15 ± 6.97	118.03 ± 5.403	134.70 ± 4.73	113.93 ± 6.17	134.21 ± 5.22	135.38 ± 5.29
SBP (mm Hg)	130.73 ± 2.13	124.53 ± .862	132.91 ± 4.40	120.84 ± 4.14	131.37 ± 4.54	134.71 ± 5.08
DBP (mm Hg)	85.70 ± 1.92	82.49 ± 2.53	87.25 ± 5.02	80.73 ± 1.60	84.82 ± 3.97	85.50 ± 4.93

Note: Values are presented as mean ± standard error (SE) for continuous variables and percentage (%) for categorical variables. CART = Concurrent Aerobic–Resistance Training; CRAT = Concurrent Resistance–Aerobic Training; COT = Control; ADEi = Average Daily Energy Intake; BMI = Body Mass Index; HOMA-IR = Homeostatic Model Assessment of Insulin Resistance; FBS = Fasting Blood Sugar; SBP = Systolic Blood Pressure; DBP = Diastolic Blood Pressure.

**Fig 2:**
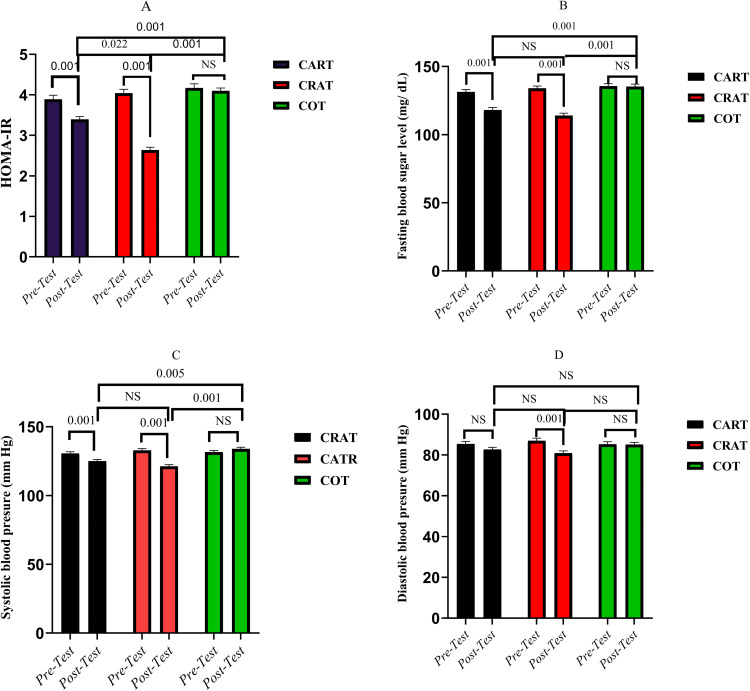
Test within and between-subject effect changes in outcomes. **Note:** *Adjusted changes in HOMA-IR* **(A)**, *fasting blood sugar* **(B)**, *systolic blood pressure* **(C)**, *and diastolic blood pressure (D) following aerobic–resistance (CART), resistance–aerobic (CRAT), and control (COT) interventions. Data were adjusted for covariates, with covariates evaluated at the following value: average daily energy intake = 2431.0959 kcal. Results are presented as mean ± SE, with significant within-group and between-group differences indicated (p < 0.05).*

The effects within subjects and pairwise comparisons are highlighted in Fig 2. Regarding HOMA-IR, notable changes were detected in CART (F = 21.912, p = .001, η² = .422), with an average reduction of 0.497 (95% CI: 0.347–0.648, p = .001), and in CRAT, which revealed a more substantial decrease of 1.401 (95% CI: 1.252–1.550, p = .001). No significant change was observed in COT (p = NS). Fasting blood glucose (FBS) also showed significant improvement in CART (F = 5.764, p = .023, η² = .161), with a mean decline of 13.249 mg/dL (95% CI: 9.474–17.024, p = .001), and even more pronounced in CRAT with a drop of 20.027 mg/dL (95% CI: 16.287–23.767, p = .001). Conversely, COT did not exhibit any significant change. For systolic blood pressure (SBP), CART indicated a considerable reduction (F = 3.621, p = .007, η² = .208) with a mean decrease of 5.613 mmHg (95% CI: 2.651–8.574, p = .001), while CRAT revealed a greater decline of 11.576 mmHg (95% CI: 8.642–14.510, p = .001). No significant effect was noted in COT. In terms of diastolic blood pressure (DBP), CART did not display a significant change, whereas CRAT showed a noteworthy reduction of 6.114 mmHg (95% CI: 2.870–9.359, p = .001). COT again did not present any significant differences.

The Table 2 and Fig 2A-2D demonstrate the between-subjects effects and pairwise comparisons. For HOMA-IR, a significant group effect was found (F = 24.460, p = .001, η² = .620), with CRAT showing greater reductions than CART (mean difference = 0.303, p = .022), and both CART (−0.493, p = .001) and CRAT (−0.796, p = .001) outperforming COT. Fasting blood glucose (FBS) also differed significantly between groups (F = 16.206, p = .001, η² = .519), with lower levels in CART (−10.747, p = .001) and CRAT (−11.459, p = .001) compared to COT, while no difference emerged between CART and CRAT. For systolic blood pressure (SBP), significant differences were observed (F = 8.968, p = .001, η² = .374), where both CART (−4.896, p = .005) and CRAT (−5.681, p = .001) showed greater reductions than COT, but not between each other. No significant between-group differences were detected for diastolic blood pressure (DBP).

**Table 2:** Test between-subject effect changes in outcomes within treatment groups.

Variables	Between-subjects effects			Pairwise comparison					
	F	Sig.^b^	η²	Treatment groups	Mean Diff.	Std. Error	Sig.^b^	95% CID	
								Lower Bound	Upper Bound
HOMA-IR	24.46	.001	.620	CART-CRAT	.303	.105	.022	.036	.571
				CART-COT	−.493	.115	.001	−.785	−.201
				CRAT-COT	−.796	.114	.001	−1.085	−.507
FBS (mg/ dL)	16.20	.001	.519	CART-CRAT	.712	2.025	NS	−4.422	5.846
				CART-COT	−10.747	2.215	.001	−16.36	−5.129
				CRAT-COT	−11.459	2.189	.001	−17.01	−5.907
SBP (mm Hg)	8.968	.001	.374	CART-CRAT	.785	1.309	NS	−2.534	4.104
				CART-COT	−4.896	1.432	.005	−8.527	−1.265
				CRAT-COT	−5.681	1.415	.001	−9.270	−2.093
DBP (mm Hg)	.610	NS	.039	CART-CRAT	.104	1.108	NS	−2.706	2.913
				CART-COT	−1.125	1.212	NS	−4.198	1.949
				CRAT-COT	−1.228	1.198	NS	−4.266	1.810

Note: The data were corrected for confounders, which were examined at an average daily energy intake (ADEi) of 2431.0959 kcal. Values are presented as adjusted means ± SE, with significant differences between groups at p < 0.05. Abbreviations: CART = Concurrent Aerobic – Resistance Training; CRAT = Concurrent Resistance – Aerobic Training; COT = Control; HOMA-IR = Homeostatic Model Assessment of Insulin Resistance; FBS = Fasting Blood Sugar; SBP = Systolic Blood Pressure; DBP = Diastolic Blood Pressure; NS = Not Significant.

## Discussion

This study investigated the effects of two concurrent training sequences, concurrent aerobic followed by resistance (CART) and resistance followed by aerobic (CRAT), to a non-exercising control group (COT) on metabolic and cardiovascular outcomes in patients with type 2 diabetes. Both exercise groups experienced significant within-group improvements in HOMA-IR, fasting blood sugar (FBS), and systolic blood pressure (SBP), but diastolic blood pressure (DBP) reductions were not substantial. Between-group analysis demonstrated that CART and CRAT outperformed COT for HOMA-IR, FBS, and SBP, indicating the efficacy of concurrent training for improving metabolic and cardiovascular performance.

Between-group comparisons indicated that both CART and CRAT significantly improved HOMA-IR relative to COT. Previous research has shown that whether aerobic exercise precedes resistance or vice versa, both sequences improve insulin sensitivity through enhanced glucose absorption and increased insulin receptor activity in patients with type 2 diabetes [8,23,24]. Additionally, CRAT showed a modestly greater reduction than CART, suggesting that performing resistance training before aerobic exercise may optimize insulin sensitivity. In this regard, combined resistance aerobic exercise training effectively improves glycemic control in male patients with type 2 diabetes [25,26]. These findings are consistent with earlier research indicating that performing resistance exercise before aerobic training increases skeletal muscle adaptations [27] which promote glucose absorption, principally through increased GLUT-4 translocation and insulin receptor sensitivity [28,29]. The greater reduction in CRAT may be related to reduced interference effects, allowing more effective activation of resistance-induced signaling pathways such as mTOR, enhancing glucose disposal [27].

There was no noticeable distinction between the two exercise sequences, and both CART and CRAT significantly decreased FBS as compared to COT. This implies that the glucose-lowering advantage of concurrent training is constant independent of sequence, even though exercise order may have a minor impact on insulin sensitivity. This suggests that while exercise order may have a little effect on insulin sensitivity, the glucose-lowering benefit of concurrent training is constant regardless of sequence. One possible explanation is that resistance training also increases muscle mass and glycogen storage capacity, while both aerobic exercise and resistance training activate complementary pathways, such as AMPK-mediated GLUT-4 translocation [30], increased insulin receptor sensitivity, and stimulation of mitochondrial biogenesis [31]. These results are in line with other research that shows that glycemic control in type 2 diabetes can be improved by combining aerobic and resistance exercise [32,33], as well as research that found comparable advantages when resistance exercise precedes aerobic training [25,34].

SBP was significantly reduced in both CART and CRAT compared to COT, with no significant difference observed between the two exercise sequences. This suggests that the blood pressure–lowering effect of concurrent training is consistent regardless of exercise order. A likely explanation is that aerobic and resistance exercise both enhance vascular function through mechanisms such as improved endothelial function [35], reduced arterial stiffness [36,37], and enhanced nitric oxide bioavailability [38,39]. These results are consistent with previous studies reporting significant reductions in SBP following combined aerobic and resistance training in type 2 diabetes patients [40]. Similarly, other research has shown comparable improvements when resistance training precedes aerobic exercise [23]. However, other studies have reported that concurrent aerobic followed by resistance exercise can produce post-exercise hypotension regardless of the exercise order in controlled hypertensive older adults [41]. Even though diastolic blood pressure in the exercise groups was significantly decreased after the exercise program, compared to before the exercise. However, there were no significant differences observed in DBP between groups, which aligns with prior findings that 12-week exercise interventions often exert minimal effects on diastolic pressure, particularly among individuals with type 2 diabetes [40,42,43]. A possible explanation is that concurrent exercise may reduce plasma endothelin-1 concentration, a vasoconstrictor and risk factor for hypertension, thereby directly contributing to reductions in SBP [44].

This study has two main limitations. First, the majority of participants were men, which may limit the generalizability of the findings and hinder the analysis of sex-related differences, largely due to challenges in recruiting female participants. Second, an intention-to-treat (ITT) analysis was not performed due to the small sample size and adherence-based inclusion criteria, and a per-protocol approach was used instead. While this allowed clearer assessment of intervention effects among completers, it may have introduced bias and reduced external validity.

## Conclusion

In conclusion, concurrent training is a safe and effective intervention for improving metabolic and cardiovascular outcomes in people with type 2 diabetes. Both CART and CRAT resulted in significant improvements in HOMA-IR, fasting blood sugar, and systolic blood pressure when compared to non-exercising controls, whereas diastolic blood pressure remained relatively stable. Importantly, CRAT showed a greater improvement in insulin sensitivity, suggesting that exercise order may have an additional effect in optimizing metabolic adaptations. These findings suggest that concurrent training has therapeutic value as an adjunct to medical care, recommending its incorporation into diabetes management regimens to enhance glucose control and reduce cardiovascular risk.

## Supporting information

S1 File CONSORT 2025 editable checklist. (DOCX)

S2 File Detail research protocol. (DOCX)
