# Supplementary material for: Impact of exercise sequence in concurrent training on insulin resistance, glycemic control, and blood pressure in Type 2 diabetes
Source: PLoS One. 2026 Jan 7;21(1):e0340587. doi: 10.1371/journal.pone.0340587 (PMC12779143; doi:10.1371/journal.pone.0340587)
Supplement: S2 File — (DOCX) [file pone.0340587.s002.docx]

**Research protocol: part 1**

**Project summary**

Type II diabetes mellitus (T2DM) is a progressive metabolic disorder associated with impaired glycemic control, insulin resistance, and elevated cardiovascular risk. Exercise is a well-established non-pharmacological therapy; however, the optimal sequencing of aerobic and resistance training in concurrent exercise programs remains unclear. Clarifying whether exercise order influences cardiometabolic adaptations is essential for refining exercise prescriptions in T2DM management.

The objective of this randomized controlled trial is to investigate the effect of exercise sequence on cardiovascular function and biochemical biomarkers in patients with T2DM. Specifically, the study compares the effects of aerobic–resistance versus resistance–aerobic training sequences on insulin resistance, glycemic control, lipid profile, and cardiovascular function.

A total of 39 men and women aged 41–60 years are recruited and randomly allocated into three groups: concurrent aerobic–resistance training (CART), concurrent resistance–aerobic training (CRAT), and a non-exercising control group (COT). Allocation is stratified by age and sex. The intervention spans 12 weeks, with supervised exercise sessions conducted three times per week. In CART, aerobic exercise precedes resistance training, whereas in CRAT, resistance exercise precedes aerobic training. The control group maintains standard care without structured exercise.

The primary outcomes include changes in cardiovascular function and biochemical biomarkers response. Secondary outcomes include anthropometric indices (BMI, body fat percentage, and waist–hip ratio).

This trial is expected to demonstrate that concurrent training improves metabolic and cardiovascular health in T2DM patients, with potential sequence-specific effects. Findings provide practical evidence for optimizing exercise prescriptions and enhancing the clinical management of T2DM

**General information**

**Protocol Title:**

Cardiovascular Function and Biochemical Biomarkers Response to Concurrent Training: Effect of Exercise Sequence on Type II Diabetic Patients – A Randomized Controlled Trial

**Protocol Identifying Number:**

The protocol was identified by Debre Markos University, Sport Science Academy (Reference No. SpScAc.IRC/03/2025). Date of approval: June 01/2025. All participants provided written informed consent before enrollment. The trial registration (Registration No: PACTR202509591505325) was completed during the intervention period due to administrative delays and limited awareness of registration requirements at the time.

**Date:**
April 10/2025

**Sponsor/Funder:**
Addis Ababa University College of Education and Language Studies,
Department of Sport Science and Physical Education
Addis Ababa, Ethiopia

**Principal Investigator:**
Mr. Friew Amare
PhD Candidate, Sport Science (Exercise and Health)
Addis Ababa University, Addis Ababa, Ethiopia
Lecturer, Debre Markos University, Debre Markos, Ethiopia
Email: [firewa6070@gmail.com] | Phone: [+251913912776]

**Co-Investigators / Collaborators (if any):**

- **Alemmebrat Kiflu**, Addis Ababa University, Ethiopia ([alemmebrat.kiflu@yahoo.com/](mailto:alemmebrat.kiflu@yahoo.com/) [alemmebrat.kiflu@aau.edu.et](mailto:alemmebrat.kiflu@aau.edu.et) )
- **Aschenaki Taddese**, Addis Ababa University, Ethiopia ([aschenakitaddese1@gmail.com/](mailto:aschenakitaddese1@gmail.com/) [aschenaki.taddese@aau.edu.et](mailto:aschenaki.taddese@aau.edu.et) )

**Research Sites:**

- **Debre Markos University, Sport Science Academy**
  *Responsibilities:* Participant recruitment, training sessions, and follow-up.

**Clinical Laboratory:**

- **Debre Markos Referral Hospital and Wudassie Diagnostic Center,**
- *Responsibilities:* Biochemical analysis, including fasting insulin, lipid profile, and related biomarkers.

**Rationale & background information**

Type 2 diabetes (T2DM) is a growing global health concern and one of the most serious issues impacting human health [1]. Affecting millions of people, it is recognized as the sixth leading cause of death worldwide [2]. Weird patterns show that prevalence is increasing not only in developed countries but also worldwide regions [3]. According to the International Diabetes Federation (IDF), Ethiopia has the largest diabetic population in Sub-Saharan Africa, with 1.96 million adults aged 20 to 79 affected [4]. Alarmingly, around 40% of these individuals were unaware of their condition [5]. The disease's economic costs has risen to $200 million annually [4]IDF (2017). IDF (2017) The lack of awareness is largely due to the gradual progression of diabetes, with early-stage symptoms often being subtle or unnoticeable, making it challenging to recognize through classic diagnostic signs [6].

Effectively tackling this issue demands a proactive approach to both prevention and treatment. As outlined in the ACSM and ADA guidelines, effective diabetes management adopts a comprehensive approach, combining pharmacological treatments with non-pharmacological strategies such as regular physical activity, dietary adjustments, and appropriate medication [7, 8].

Considering factors such as efficacy, cost, potential side effects, weight gain, comorbidities, and the risk of hypoglycemia, patients should prefer non-pharmacological approaches [9]. Similarly, different researchers demonstrate regular physical exercise is a fundamental component of T2DM management, demonstrably improving glycemic control and overall well-being [10-13]. Specifically, resistance training (RT) has been shown to promote skeletal muscle growth, which contributes to fat reduction [14], and improved metabolic outcomes, including lower fasting plasma glucose (FPG), enhanced insulin sensitivity, and better glucose tolerance in diabetic patients [15]. Additionally, RT benefits lipid regulation by increasing HDL-c levels and reducing triglycerides (TG), making it an effective intervention for T2DM management [16]. Similarly, a meta-analysis and systematic review reviled that, aerobic exercise has demonstrated positive effects on markers of metabolic syndrome (MetS), including reduced fasting glucose, higher HDL-c levels, and lower TG levels [17, 18], moreover, it helps reduce systolic blood pressure (SBP), diastolic blood pressure (DBP), resting heart rate (RHR), fasting blood sugar (FBS), and body mass index (BMI), while improving oxygen saturation (SpO2) compared to the control group in individuals with T2DM [19].

While both aerobic and resistance exercise have their benefits, concurrent training (CT), which incorporates the special advantages of both, is typically recommended for the treatment of type 2 diabetes. There is evidence from numerous studies that it works. Glycated hemoglobin (HbA1c), body fat, peak oxygen uptake, cholesterol, and insulin resistance (HOMA-IR) were all found to significantly improve in people receiving concurrent aerobic and resistance exercise, according to Bassi, Mendes [20]. Similarly, Ambelu and Teferi [21] found that a combined aerobic-strength program significantly lowered body composition measures, blood pressure, and fasting blood glucose. However, despite the recognized benefits of CT, research on the optimal sequencing of aerobic and resistance exercises within this training approach remains limited and inconclusive.

Molecular bases the sequence of endurance and resistance exercise in concurrent training significantly influences responses due to AMPK-mTORC1 interferences. The aerobic-first sequence (AE-RE) enhances mitochondrial biogenesis, fat oxidation, and cardiovascular efficiency but may lead to residual fatigue, impair strength performance, and inhibit mTORC1 (mammalian target of rapamycin complex 1), limiting hypertrophy and strength [22]. Moreover, glycogen depletion from aerobic exercise reduces the intensity of subsequent resistance training, ultimately diminishing its effectiveness [23]. Conversely, the resistance-first sequence (RE-AE) maximizes strength and hypertrophy gains by preserving mTORC1 activation and neuromuscular efficiency, and research has also demonstrated that this training sequence had no impact on aerobic capacity [24, 25], however, Doma, Deakin [26] demonstrated that a resistance-first sequence may impair endurance performance due to resistance-induced fatigue and increased cardiovascular strain.

This concept is still inconclusive, while some studies indicate that the sequence of exercises in a CT program may affect cardiovascular and metabolic outcomes. For instance, Delgado-Floody, Soriano-Maldonado [27] displayed (AT + RT) significant decreases in waist circumferences (WC), but not for SBP, DBP, FPG, HDL-c and TG, all *p*>0.05 comparing to RT + AT among in women with severe/morbid obesity. Likewise, Kobayashi, Long [28] observed no significant changes in HbA1c when concurrent training was performed in an opposite sequence, whereas Church, Blair [29] observed significant changes in HbA1c with aerobic resistance sequence of exercises, despite improvements in VO2 max and fat mass.

Regarding lipid profiles, among non-diabetic patients, Flores-Moreno, Del [30] observed significant improvements in BMI, cholesterol, and glucose regulation when resistance training was followed by aerobic exercise among sedentary obese individuals. Conversely, Amare, Alemu [31] highlighted that an aerobic-first approach led to significant enhancements in lipid profiles and glycemic control in overweight and obese adults. Additionally, Azarbayjani, Abedy [32] noted improvements were observed in anthropometric measures and insulin resistance when aerobic exercise preceded resistance training, though no significant changes were observed in lipid profiles among sedentary adults.

These findings’ variation suggests that the sequence of aerobic and resistance exercises within concurrent training programs needs further research to identify the optimal sequence of exercises to maximize health benefits for Type II diabetic patients. Some studies suggest that performing resistance training before aerobic exercise enhances in healthy population fat oxidation and muscle glucose uptake, optimizing metabolic adaptations [33] and improves muscular strength and overall physical fitness [24, 34]. In contrast, an aerobic-first approach improves reducing visceral fat and inducing anabolism after exercise [35] and potentially inducing muscle fatigue [36], which could compromise resistance training performance [37]. Nevertheless, a recent systematic review and meta-analysis by Canli and Aldhahi [38] found no significant effect of exercise order on overall workout effectiveness or physiological adaptations. These conflicting findings highlight a critical gap in understanding the influence of exercise sequence on health outcomes, particularly in diabetic patients, underscoring the need for further research to determine the optimal exercise order for maximizing health benefits.

A key unanswered question is: How does the sequence of exercises in a CT program influence metabolic, cardiovascular, and anthropometric outcomes in individuals with Type II diabetes? And also, studies have highlighted the importance of conducting future research to compare the effects of different exercise sequences within CT for this special population [20, 39]. Moreover, previous research on CT for diabetic patients has not sufficiently accounted for diet as a confounding factor, which can greatly influence cardiovascular function and biochemical biomarker outcomes [40]. Understanding how exercise order impacts key anthropometric measures, cardiovascular function, and biochemical biomarkers is essential for optimizing treatment strategies.

To effectively address this gap in the literature, the study will evaluate and compare the order of exercise in concurrent training on the influence of anthropometric measures, biochemical markers, and cardiovascular function in middle-aged men with type 2 diabetes. To ensure accurate assessment of the exercise program's health impacts, participants will adhere to a strict dietary monitoring protocol [41].

**References**

1. Ginter, E. and V. Simko, *Type 2 Diabetes Mellitus, Pandemic in 21st Century*, in *Diabetes: An Old Disease, a New Insight*, S.I. Ahmad, Editor. 2013, Springer New York: New York, NY. p. 42-50.

2. Jain, S. and S. Saraf, *Type 2 diabetes mellitus—Its global prevalence and therapeutic strategies.* Diabetes & Metabolic Syndrome: Clinical Research & Reviews, 2010. **4**(1): p. 48-56.

3. Liu, J., et al., *Trends in the incidence of diabetes mellitus: results from the Global Burden of Disease Study 2017 and implications for diabetes mellitus prevention.* BMC Public Health, 2020. **20**(1): p. 1415.

4. IDF, *IDF diabetes atlas 10th edition*. 2021.

5. Solomon, H., et al., *Landscape Assessment of Diabetes Mellitus in Ethiopia.* 2023.

6. Edmonds, M.E. and A.V. Foster, *Managing the diabetic foot*, ed. r. edition. 2014: John Wiley & Sons.

7. Colberg, S.R., et al., *Physical activity/exercise and diabetes: a position statement of the American Diabetes Association.* Diabetes care, 2016. **39**(11): p. 2065.

8. White Jr, J.R., *A brief history of the development of diabetes medications.* Diabetes spectrum: a publication of the American Diabetes Association, 2014. **27**(2): p. 82.

9. Chaudhury, A., et al., *Clinical Review of Antidiabetic Drugs: Implications for Type 2 Diabetes Mellitus Management.* Front Endocrinol (Lausanne), 2017. **8**: p. 6.

10. Kanaley, J.A., et al., *Exercise/physical activity in individuals with type 2 diabetes: a consensus statement from the American College of Sports Medicine.* Medicine and science in sports and exercise, 2022. **54**(2): p. 353.

11. Teixeira-Lemos, E., et al., *Regular physical exercise training assists in preventing type 2 diabetes development: focus on its antioxidant and anti-inflammatory properties.* Cardiovascular Diabetology, 2011. **10**(1): p. 12.

12. Kirwan, J.P., J. Sacks, and S. Nieuwoudt, *The essential role of exercise in the management of type 2 diabetes.* Cleve Clin J Med, 2017. **84**(7 Suppl 1): p. S15-s21.

13. Umpierre, D., et al., *Physical activity advice only or structured exercise training and association with HbA1c levels in type 2 diabetes: a systematic review and meta-analysis.* Jama, 2011. **305**(17): p. 1790-9.

14. Hovanec, N., et al., *Resistance training and older adults with type 2 diabetes mellitus: strength of the evidence.* Journal of aging research, 2012. **2012**.

15. Lee, J., D. Kim, and C. Kim, *Resistance training for glycemic control, muscular strength, and lean body mass in old type 2 diabetic patients: a meta-analysis.* Diabetes Therapy, 2017. **8**: p. 459-473.

16. Barzegari, A. and H. Amouzad Mahdirejei, *Effects of 8 weeks resistance training on plasma vaspin and lipid profile levels in adult men with type 2 diabetes.* Caspian J Intern Med, 2014. **5**(2): p. 103-8.

17. Wewege, M.A., et al., *Aerobic, resistance or combined training: A systematic review and meta-analysis of exercise to reduce cardiovascular risk in adults with metabolic syndrome.* Atherosclerosis, 2018. **274**: p. 162-171.

18. Kelley, G. and K. Kelley, *Effects of aerobic exercise on lipids and lipoproteins in adults with type 2 diabetes: a meta-analysis of randomized-controlled trials.* Public health, 2007. **121**(9): p. 643-655.

19. Ezema, C.I., et al., *The Effect of an Aerobic Exercise Programme on Blood Glucose Level, Cardiovascular Parameters, Peripheral Oxygen Saturation, and Body Mass Index among Southern Nigerians with Type 2 Diabetes Mellitus, Undergoing Concurrent Sulfonylurea and Metformin Treatment.* Malays J Med Sci, 2019. **26**(5): p. 88-97.

20. Bassi, D., et al., *Potential Effects on Cardiorespiratory and Metabolic Status After a Concurrent Strength and Endurance Training Program in Diabetes Patients - a Randomized Controlled Trial.* Sports Med Open, 2015. **2**: p. 31.

21. Ambelu, T. and G. Teferi, *The impact of exercise modalities on blood glucose, blood pressure and body composition in patients with type 2 diabetes mellitus.* BMC Sports Science, Medicine and Rehabilitation, 2023. **15**(1): p. 153.

22. Apró, W., et al., *Resistance exercise-induced S6K1 kinase activity is not inhibited in human skeletal muscle despite prior activation of AMPK by high-intensity interval cycling.* Am J Physiol Endocrinol Metab, 2015. **308**(6): p. E470-81.

23. Fyfe, D.J. Bishop, and N.K. Stepto, *Interference between concurrent resistance and endurance exercise: molecular bases and the role of individual training variables.* Sports Med, 2014. **44**(6): p. 743-62.

24. Murlasits, Z., Z. Kneffel, and L. Thalib, *The physiological effects of concurrent strength and endurance training sequence: A systematic review and meta-analysis.* J Sports Sci, 2018. **36**(11): p. 1212-1219.

25. Vikestad, V. and T. Dalen, *Effect of Strength and Endurance Training Sequence on Endurance Performance.* Sports (Basel), 2024. **12**(8).

26. Doma, K., G.B. Deakin, and D.J. Bentley, *Implications of Impaired Endurance Performance following Single Bouts of Resistance Training: An Alternate Concurrent Training Perspective.* Sports Medicine, 2017. **47**(11): p. 2187-2200.

27. Delgado-Floody, P., et al., *The Effects of Two Different Concurrent Training Configurations on Markers of Metabolic Syndrome and Fitness in Women With Severe/Morbid Obesity: A Randomised Controlled Trial.* Front Physiol, 2021. **12**: p. 694798.

28. Kobayashi, Y., et al., *Strength training is more effective than aerobic exercise for improving glycaemic control and body composition in people with normal-weight type 2 diabetes: a randomised controlled trial.* Diabetologia, 2023. **66**(10): p. 1897-1907.

29. Church, T.S., et al., *Effects of aerobic and resistance training on hemoglobin A1c levels in patients with type 2 diabetes: a randomized controlled trial.* Jama, 2010. **304**(20): p. 2253-62.

30. Flores-Moreno, P.J., et al. *The effect of concurrent training on glucose, lipid profile, liver enzymes, and lipid peroxidation in young men*. 2024.

31. Amare, F., et al., *Effects of aerobic, resistance, and combined exercise training on body fat and glucolipid metabolism in inactive middle-aged adults with overweight or obesity: a randomized trial.* BMC Sports Science, Medicine and Rehabilitation, 2024. **16**(1): p. 189.

32. Azarbayjani, M.A., et al., *Effects of combined aerobic and resistant training on lipid profile and glycemic control in sedentary men.* International Medical Journal, 2014. **21**: p. 132-136.

33. Kang, J., et al., *Effect of preceding resistance exercise on metabolism during subsequent aerobic session.* Eur J Appl Physiol, 2009. **107**(1): p. 43-50.

34. Li, Z., et al., *Impact of Sequence in Concurrent Training on Physical Activity, Body Composition, and Fitness in Obese Young Males: A 12-Week Randomized Controlled Trial.* Journal of Exercise Science & Fitness, 2025.

35. Wu Min, et al., *Effect of concurrent training sequences on body composition and hormone response: a Meta-analysis.* Chinese Journal of Tissue Engineering Research, 2022. **26**(8): p. 1305-1312.

36. Cadore, et al., *Strength prior to endurance intra-session exercise sequence optimizes neuromuscular and cardiovascular gains in elderly men.* Exp Gerontol, 2012. **47**(2): p. 164-9.

37. Coffey, V.G. and J.A. Hawley, *Concurrent exercise training: do opposites distract?* The Journal of Physiology, 2017. **595**(9): p. 2883-2896.

38. Canli, U. and M.I. Aldhahi, *The physiological and physical benefits of two types of concurrent training: a randomized controlled trial.* BMC Sports Science, Medicine and Rehabilitation, 2024. **16**(1): p. 8.

39. Banitalebi, E., et al., *Comparing the Effects of Eight Weeks of Combined Training (Endurance and Resistance) in Different Orders on Inflammatory Factors and Adipokines Among Elderly Females.* Women’s Health Bulletin, 2016. **3**(2): p. 1-10.

40. Tuso, P., *Prediabetes and lifestyle modification: time to prevent a preventable disease.* Perm J, 2014. **18**(3): p. 88-93.

41. Matthews, C.E., et al., *Best practices for using physical activity monitors in population-based research.* Med Sci Sports Exerc, 2012. **44**(1 Suppl 1): p. S68-76.

**Study goals and objectives**

The primary aim of this study is to evaluate the effects of aerobic–resistance versus resistance–aerobic exercise sequences within concurrent training on cardiovascular function and biochemical biomarkers in patients with Type 2 diabetes, based on the evidence gaps identified in a prior systematic review and meta-analysis.

**Study design**

This study adopts a two-phase sequential research design to investigate the impact of exercise sequence in concurrent training on patients with type II diabetes. Phase I involved conducting a systematic review and meta-analysis titled “Effect of concurrent continuous aerobic and short rest resistance exercise training on metabolic biomarkers in type 2 diabetes patients: a systematic review and meta-analysis.” This review aimed to summarize and synthesize existing evidence on the effects of concurrent training on metabolic outcomes such as HbA1c, insulin resistance, and lipid profile. The review identified both clinical and methodological gaps in the current literature, with a notable lack of evidence on the role of exercise sequence: specifically, whether performing aerobic training before or after resistance training and controlling confounding variable has differential effects on key metabolic and cardiovascular biomarkers in this population.

Building on the findings from the systematic review, Phase II employs a parallel-group randomized controlled trial (RCT) to address the gaps identified. The trial focuses on the sequence of exercise as a potentially important but under-researched variable in concurrent training programs for individuals with type II diabetes. Participants will be randomly allocated in equal numbers to one of three groups: the CART group (Concurrent Aerobic then Resistance Training), the CRAT group (Concurrent Resistance then Aerobic Training), and a wait-list control group (COT). Randomization will follow a 1:1:1 ratio and will be stratified by sex and age, with participants grouped into four age categories: 41–45 years, 46–50 years, 51–55 years, and 56–60 years. To reduce allocation bias, the randomization process will be overseen by the personal trainer and the principal researcher, while outcome assessments will be conducted by independent data collectors blinded to group assignment.

The intervention will last for 12 weeks, with participants in the CART and CRAT groups engaging in supervised concurrent training sessions for one hour per day at 5:00 PM on Tuesdays, Thursdays, and Sundays. Although the types of exercises will be identical between the two intervention groups, the order of aerobic and resistance training will differ. The researcher will monitor and compare changes in anthropometric measures, cardiovascular function, and biochemical biomarkers across the groups through pre- and post-intervention assessments. This two-phase design ensures that the RCT is grounded in a comprehensive evidence base and targets specific unanswered questions identified in the literature, thereby enhancing both the scientific rigor and practical relevance of the study.

**Methodology**

**Method and Procedures of Data Collection**

The data collection for this study will be conducted under carefully controlled environmental and procedural conditions to ensure consistency and accuracy of results. Every subject will be assessed for the same variables at the same time of day to minimize variations, which can significantly influence physiological responses such as cardiovascular function and biochemical biomarkers. The experimental room will maintain a relatively constant air humidity and temperature to prevent external factors, such as fluctuating environmental conditions, from affecting the participants' responses. This consistency in timing and environment is crucial for reducing potential variability in the data and ensuring that any observed effects can be attributed to the exercise interventions rather than external factors.

Additionally, all research and laboratory staff involved in the data collection will be rigorously trained and instructed to adhere to specific protocols to avoid influencing the participants during the assessments. They will be required to remain still and silent throughout the procedures, reducing any potential distractions or stressors for the participants.

**Measurement of biochemical variables**

Blood samples will be drawn from the antecubital vein both before and after the 12-week exercise training period. 48 hours after the most recent training session, blood samples will be taken to minimize the acute effects of exercise and ensure accurate results. In addition, participants must refrain from eating or drinking alcohol and coffee for 12 hours before the blood sample test, as well as avoid consuming alcohol or eating a high-fat diet.

The blood samples will be collected by a medical laboratory technician from Debre Markos Referral Hospital, who was trained in the procedure. A trained technician will utilize a venipuncture to extract blood from the antecubital vein while the participants will be seated. These samples will then be analyzed to evaluate various biochemical parameters. With some careful planning and preparation, a single blood draw will be used to acquire the sample to evaluate all of the biomarkers (HbA1c, Insulin Sensitivity, Glucose Tolerance, HDL, LDL, TC and Triglycerides) based on preplanned standard operating procedures.

**Blood collection tools**

- Blood collection tubes (e.g., EDTA, serum separator tubes).
- Sterile needles and syringes
- Alcohol swabs
- Tourniquet
- Cotton balls
- Sharps disposal container

**Venipuncture Procedure**

Position the patient comfortably in a seated position, with the arm fully extended and the palm facing upward. Select an accessible vein, typically in the antecubital fossa, while avoiding areas with bruises, scars, or inflammation. Apply a tourniquet 3–4 inches above the venipuncture site and check for an easily accessible vein. Clean the site thoroughly using an alcohol swab in a circular motion, starting from the center and moving outward, and allow it to air dry completely. Press the vein by holding the skin with your thumb below the puncture site, and insert the needle bevel side up at a 15–30-degree angle.

For sample collection, follow the correct order of draw to avoid cross-contamination by collecting blood in a serum separating tube (SST) for the fasting insulin, lipid profile. Ensure sufficient blood volume is collected for all tests, typically 5 mL per tube for serum tests and 2–3 mL for HbA1c. Invert the tubes as needed for proper additive mixing, and label each tube immediately with the patient’s name, date of birth, date, and time of collection.

Since Debre Markos lacks a facility for fasting insulin level tests, the blood samples will be securely transported to the Wudassie Diagnostic Center in Addis Ababa for analysis. After collection, the fasting insulin sample will be placed in a serum separator tube (SST) and immediately labeled with the patient's information, date of birth, and time of collection. The sample will be stored in a cool, insulated container with an ice pack to maintain its integrity during transport. The sample should be delivered promptly to the diagnostic center to ensure accurate results, ideally within 24 hours. Upon arrival at the laboratory, the sample will be processed according to standard procedures for insulin level testing.

The Enzyme- Linked Immunosorbent Assay (ELISA) assay type China (Shakil-Ur-Rehman et al., 2017) will be used for the fasting insulin test assays.

The homeostasis model of insulin resistance (HOMA-IR index), which is the product of glucose and insulin concentrations divided by a factor, will be used to quantify insulin resistance (Vogeser et al., 2007). The HOMA-IR index will be calculated as:

$$HOMA-IR=\frac{fasting serum glucose (mg.\mathrm{dL}^{-1})*fasting serum insulin(\mu U.{mL}^{-1})}{405}$$

*Equation 4* *Homeostasis model of insulin resistance equation*

**Measurement of cardiovascular function variables**

**Blood pressure**

The procedure for accurately measuring blood pressure and arterial stiffness using via an automated Sphygmocor XCEL device (AtCor Medical, CardieX, Sydney, Australia) (De la Torre Hernández et al., 2021), was adapted from Mengistu et al. (2025).

**Materials Required**

- SphygmoCor XCEL device
- The appropriate brachial cuff will be selected based on arm circumference: dark blue for 22-33 cm, maroon for 31-40 cm, and brown for 38-50 cm.
- A stable and flat table and comfortable bench.

**Preparation**

- **Participant Preparation:**
  - Instruct the participant to avoid caffeine, alcohol, and strenuous exercise for at least 30 minutes prior to measurement.
  - Make sure the participant has been positioned ready and lying on the catheterization laboratory table for at least 5 minutes before starting the procedure.
  - Ask the participant to remove any clothing that may obstruct the placement of the cuff on the upper arm.

**Procedure**

- **Cuff Placement:**
  - Place the cuff on the participant’s left upper arm, positioning it so that the artery marker aligns with the brachial artery.
  - Ensure the cuff is snug but not excessively tight, and the lower edge is approximately 2-3 cm above the elbow crease.
- **Positioning:**
  - Position the arm with the cuff at heart level for accurate readings. Use cushioning if needed to maintain proper positioning.
- **Initiating the Measurement:**
  - Begin the measurement by activating the SphygmoCor XCEL system.
  - The device will automatically record the brachial blood pressure (SBP and DBP) during cuff inflation and deflation.
- **Instructions During Measurement:**
  - Instruct the participant to remain still and refrain from speaking or moving during the cuff inflation and waveform measurement phases.
- **Post-Measurement**
- Remove the cuff and assist the participant to sit up if needed.
- Record the measurements displayed by the device:
  - Brachial SBP and DBP
  - Resting heart rate
- Conduct the measurement three times to ensure accuracy, adhering to standard protocols, and use the average of the remaining two readings.

**Covariate variable (dietary assessment)**

While nutrition is not a primary focus of the study, it should be monitored due to its potential impact on the dependent variable. Therefore, we will track the average daily calorie intake through face-to-face interviews. We will be utilized a 24-hour interactive personal interview with multiple passes (Quick list, Forgotten foods, Time & occasion, Detail cycle and Final probe) that was developed and validated for use in developing countries (Blanton et al., 2006; Gibson & Ferguson, 2008) (APPENDIX B). This approach employs five steps:

- Step 1: Quick list (a list is made of foods and drinks consumed in the last 24 hours)
- Step 2: Forgotten foods (questions are asked to identify any foods that may have been overlooked in Step 1)
- Step 3: Time & occasion (the time and occasion of each food item are recorded)
- Step 4: Detail cycle (each food's detailed description, quantity, and any additions are documented)
- Step 5: Final probe (a last check is done to ensure no other foods or drinks were consumed in the past 24 hours)

The 24-hour food frequency data collect on three non-consecutive two weekdays and one weekend days (Baranowski, 2012; Buttriss et al., 2017): from Monday 6 p.m. to Tuesday 5 p.m., Wednesday 6 p.m. to Thursday 5 p.m., and Saturday 6 p.m. to Sunday 5 p.m. It is better to choose non-consecutive days, as this helps to capture a broader range of variability in an individual's diet (FAO, 2018). We will apply the Ethiopian food composition table (EHNRI, 1981; EHNRI, 1998) to estimate nutrient and energy levels from dietary data. The names of foods and drinks, their descriptions, cooking methods, and amounts from 24-hour periods will be coded and submitted to the NutriSurvey200 (Feyesa et al., 2020). After determining the frequency of consumption per day, we will use the product sum approach to determine daily food intake. Daily food intake = ∑ (food item's stated consumption frequency, translate to times per day) * (portion size ingested of that food). The daily average energy intake will also determine as follows: ADEi = ∑daily food intake/number of data collected days.

**Exercise Training Protocol**

The training regimen for the concurrent training will consist of 36 sessions of individually supervised work, three times a week for 12 weeks on alternating days (Tuesday, Thursday, and Sunday) (APPINDEX VII). Every training session will be conducted under the strict observation of fitness experts. Each 70-minute session had a 5-minute warm-up, a 60-minute main training session (30 aerobic and 30 resistance), and a 5-minute cool-down. We will use the American College of Sports Medicine's recommendation for type II diabetic individuals served as the basis for the exercise programs (ACSM, 2020).

To minimize biased influence during the main training process, a one-week familiarization period will be implemented beforehand. This familiarization will involve easily adjustable intensity aerobic exercises on a treadmill, such as walking and jogging, as well as low-load resistance exercises that participants can perform for 10–15 repetitions without experiencing fatigue.

The RT program focused on the body's major muscle groups in accordance with recommendations from the American Diabetes Association (Sigal et al., 2006). Exercises that will be done include abdominal curl, standing plantar flexion and squatting with body weight and free weights, machine leg press, neutral rowing, machine bicep curl, triceps pulley, and machine bench press (vertical press) exercise using a multi-gym, leg press machine, and dual adjustable pulley (Cybex International, Medway, Massachusetts). They will be performed between 50 to 85 percent of the estimated 1-RM in line with (Liguori & Medicine, 2020; Mager et al., 2008). Workouts using the circuit form of resistance training (RT) will be employed with intervals of 15–20s between sets, consisting of one - three sets of 10-15 repetitions to near fatigue per set with a 30 to 90 seconds break in between to improve muscle strength (Liguori & Medicine, 2020). To ensure steady repetitions throughout the training program, the weight lifted will be increased gradually.

To determine protocol loads, the 1-RM test will be applied by gradually increasing resistance until the volunteer succeeded in performing no more than one repetition. Start with a warm-up that includes a small weight that is roughly 40–60% of the perceived maximum load in order to calculate each individual's 1-RM. To make sure the muscles are prepared for the subsequent section of the test, give everyone a minute to relax after finishing the warm-up. provide the individual 12–15 repetitions after increasing the weight to a moderate load (60–80% of the perceived maximum). This set should be difficult, but not impossible. Rest for one to two minutes after finishing this set.

After establishing a reasonable weight, encourage the subject to do up to 10 repetitions with a 10% increase in load. Allow the individual an additional one to two minutes of break before continuing if they are able to perform ten or more repetitions with the new weight. Keep an eye on the participant throughout this rest period to make sure they aren't exerting themselves excessively. Increase the weight by 10% again and have them try repetition with the larger load if they are able to finish the set of 10 or more reps. After that, the resistance will be gradually raised until the individuals could only complete each exercise nine repetition or less. Reaching the target number of repetitions in between 3 and 6 tries is the aim of increasing the resistance. Three minutes of rest are permit in between each particular exercise, and two minutes will be permitted between each try. Brzycki 1-RM prediction equation(Brzycki, 1993) will then use to estimate the 1-RM based on the resistance and repetitions recorded on the last try. The mathematical expression for the equation is 1RM = W/ [102.78– 2.78(R)]/100, where R is the maximum number of repetitions and W is the weight was using (Abdul-Hameed et al., 2012).

The aerobic workout will involve using a Cybex treadmill (Cybex Corporation, Ronkonkoma, New York) (McNamara & Stearne, 2013) at moderate intensity (40–59% HRR) to vigorous intensity (60–89% HRR) (Liguori & Medicine, 2020).

We will use the heart rate reserve (HRR) approach, which is based on the Karvonen formula, to determine the target heart rate (THR) in order to manage the intensity of the exercise (Yabe et al., 2021). This approach is appropriate for a broad spectrum of adult fitness levels (ACSM's, 2013a). The following is the formula:

$$\mathrm{THR}=HR rest + [(HR max - HR rest) \times Intensity]$$

*Equation 6 HRR approach Karvonen target heart rate formula*

Whereas:

- HR max = 220 – age
- RH: expressed the number of beats per minute (bpm) at rest.
- Intensity: the decimal representation of the desired level of exercise intensity (e.g., 0.40 for 40%, 0.60 for 60%).

The Polar H7 heart rate monitor (Polar Electro, Kempele, Finland) (Hernández-Vicente et al., 2021) will be used to continually track the participants' target heart rates as they exercise. To giving real-time feedback, this device makes sure that the intensity of the workout stays within the predetermined target heart rate range.

There will be resistance and aerobic workouts within the concurrent resistance-aerobic (CRAT) and concurrent aerobic-resistance (CART) groups. The sequence in which the resistance or aerobic components will be done first and second is the only distinction between the two training regimens (CRAT and CRAT). Every training session, there will be a 5-minute recovery and transition period in between the two training programs (aerobic and resistance components).

To ensure the detection of any adverse events, heart rate, blood pressure, and blood glucose levels will be monitored before and after each exercise session, particularly if a medical professional observes unusual or concerning patterns. It would not be possible to incorporate a placebo therapy that mimicked exercise in this investigation (Arora et al., 2023). Rather, a control group will be created in which individuals continue with their regular care regimen while abstaining from any regular exercise program. This makes it possible to compare the impact of the training program with the individuals' current diabetes control techniques.

**Safety considerations**

The safety and well-being of research participants are of paramount importance throughout the study. All procedures will be conducted with strict adherence to ethical standards and institutional guidelines to minimize risk and ensure participant protection. The research protocol includes detailed measures for monitoring, recording, and reporting any adverse events, whether physical, psychological, or social. These events will be promptly addressed and followed up to ensure resolution and participant support.

Participants will be informed of potential risks during the consent process, including the possibility of discomfort or emotional distress, even from seemingly minimal interventions such as questionnaires. Trained personnel will supervise all exercise sessions to reduce the risk of injury, and medical clearance will be obtained before participation. Emergency procedures and referral pathways will be in place should any health concerns arise during the intervention.

Confidentiality and data protection will be rigorously maintained, and participants will have the right to withdraw at any time without penalty. These safeguards aim to uphold the dignity, safety, and autonomy of all individuals involved in the study.

**Follow-up**

The research protocol includes a structured follow-up plan to ensure ongoing participant safety and support beyond the active data collection phase. All participants will be monitored throughout the 12-week intervention period, with additional follow-up extending four weeks post-intervention to identify and address any delayed adverse events or health concerns.

In cases where adverse events occur, participants will receive appropriate medical attention and continued monitoring until resolution. Follow-up procedures include scheduled check-ins via phone or clinic visits, documentation of health status, and referral to healthcare providers when necessary.

This extended follow-up ensures that any physical or psychological effects related to the intervention are responsibly managed, reinforcing the study’s commitment to participant welfare and ethical research conduct.

**Data management and statistical analysis**

All data will be systematically coded and entered into a secure, password-protected database for computer-assisted analysis. Data handling procedures include double-entry verification and regular monitoring to ensure accuracy, completeness, and consistency. Any discrepancies or spurious entries will be flagged and resolved through cross-checking with source documents.

Statistical analysis will be performed using SPSS version 27. The primary analytical approach is Repeated Measures Analysis of Covariance (RM ANCOVA), which allows for the evaluation of time-dependent changes across intervention groups while controlling for dietary practice as a covariate. This adjustment accounts for individual variability in nutritional habits that may influence metabolic outcomes.

The model will assess:

- **Main effects of time** (pre- vs post-intervention),
- **Main effects of group** (CART, CRAT, COT),

Assumptions of normality, homogeneity of variance, and sphericity will be tested prior to analysis. Violations will be corrected using appropriate statistical adjustments (e.g., Greenhouse-Geisser correction). The level of significance is set at *p* < 0.05, and partial eta squared (η²) will be reported to indicate effect sizes.

The sample size of 39 was determined through power analysis, targeting 80% power to detect moderate effect sizes in insulin resistance and cardiovascular outcomes, with a 5% significance level and allowance for 10% attrition.

**Quality assurance**

This study's quality control method includes a number of crucial steps to guarantee correctness and dependability. Participants in each group will first be thoroughly matched to the intervention groups and control group according to important clinical and demographic traits, like gender and age. To guarantee comparability across all groups, stratification will be used. The International Physical Activity Questionnaire (IPAQ) will be utilized to assess physical activity levels among control group participants. Data will be collected through interviewer-administered IPAQ during weekly check-ins. Participants in the control group who report engaging in over 150 minutes of moderate physical activity per week will be excluded from the study.

Equipment will be calibrated before each test session, and all biochemical, cardiovascular, and anthropometric index measurements will follow established testing procedures. All participants will take tests at the same time of day to reduce variability. To avoid measurement bias, the test data collector and analyst will be masked to group assignments. All testing personnel will also receive extensive training to guarantee the capability to follow the set procedures.

In order to ensure that participants complete exercises with the right form and intensity, trained exercise physiologists will oversee all training sessions. Both attendance and any sessions that are missed will be noted. Lastly, weekly progress checks will be conducted to make sure participants are performing the exercises at the recommended volume and intensity.

**Expected outcomes of the study**

This study is expected to advance scientific understanding of how exercise sequencing—specifically aerobic-resistance versus resistance-aerobic training affects cardiometabolic health in individuals with Type II diabetes mellitus (T2DM). By controlling for dietary practice and using robust statistical methods, the findings will clarify whether the order of exercise modalities influences improvements in insulin sensitivity, glycemic control, lipid profile, and cardiovascular function.

The results will be disseminated through peer-reviewed publications and conference presentations, contributing to the academic literature on diabetes management and exercise physiology. Beyond scholarly impact, the findings have practical implications for clinical care: they will inform evidence-based exercise prescriptions tailored to the needs of T2DM patients, enhancing the effectiveness of non-pharmacological interventions.

At the health system level, the study supports integration of structured, sequence-aware exercise programs into diabetes care protocols, particularly in resource-limited settings like Ethiopia. If sequence-specific benefits are confirmed, training guidelines for physiotherapists and diabetes educators can be refined to maximize patient outcomes.

From a policy perspective, the evidence may guide health authorities in developing or updating diabetes management strategies, promoting exercise as a cost-effective tool to reduce disease burden and improve quality of life. The study also aligns with broader public health goals by encouraging lifestyle-based interventions that are scalable, sustainable, and culturally adaptable.

**Dissemination of results and publication policy**

The results of this trial will be disseminated through multiple channels to maximize scientific, clinical, and community impact. Findings will first be submitted for publication in peer-reviewed scientific journals specializing in diabetes, exercise science, and clinical health. Presentations will also be made at national and international scientific conferences.

In addition to academic dissemination, results will be communicated directly to the participating patients and their families in a clear and accessible manner. Community-level dissemination will be conducted through local health centers, diabetes associations, and public forums to ensure broader awareness of the benefits of exercise interventions. Where appropriate, findings will also be shared with relevant policy-making bodies, including the Ministry of Health and regional health bureaus, to support evidence-based recommendations for diabetes management and prevention strategies.

Publication policy follows international research ethics standards. The principal investigator will take the lead in drafting and submitting manuscripts. Co-investigators who contribute substantially to the design, implementation, analysis, or interpretation of results will be included as co-authors. Data collectors, laboratory staff, and other contributors will be acknowledged appropriately in publications and presentations. No publications arising from this study will be submitted without the agreement of the research team.

**Duration of the project**

**Project Timeline**

The protocol specifies the anticipated duration of each phase of the project along with a detailed month-by-month schedule of activities. The overall project spans 9 months, beginning from January and ending with dissemination of results.

- January – February (Phase One: Systematic Review):
  - Objective: To summarize and synthesize existing evidence on the impact of concurrent training on metabolic biomarkers in individuals with Type 2 Diabetes Mellitus.
  - Activities:
    1. Conduct a comprehensive literature search across databases.
    2. Screen studies for eligibility based on pre-defined inclusion and exclusion criteria.
    3. Extract and organize data on concurrent training interventions, metabolic outcomes, and study characteristics.
    4. Critically appraise study quality and risk of bias.
    5. Identify gaps in current evidence to inform the experimental intervention.
- March – April (Phase Two: Intervention Protocol Preparation):
  - Develop detailed exercise intervention protocols for CART and CRAT groups.
  - Prepare training manuals, standard operating procedures, and data collection tools.
  - Train research staff on protocol implementation and data management.
- May: Submission and approval of ethical clearance from relevant institutional review boards.
- June – August (Phase Three: Intervention Implementation):
  - Recruit participants, conduct screening and baseline assessments.
  - Implement the 12-week supervised concurrent training program (CART and CRAT groups).
  - Monitor adherence, record session attendance, and collect ongoing data.
- September (Phase Four: Post-Intervention Assessment and Follow-Up):
  - Conduct post-intervention measurements of metabolic, cardiovascular, and anthropometric outcomes.
  - Clean and organize collected data for analysis.
- September - October: Data analysis, interpretation, and preparation of manuscripts.
- September - January: Dissemination of results to participants, the community, scientific audiences, and policy makers.

**Problems anticipated**

While the study is designed to be feasible within the proposed 12-week timeline and allocated budget, several challenges may arise during implementation. One key concern is participant retention, particularly in the intervention groups, due to time constraints, transportation issues, or health-related setbacks. To address this, flexible scheduling, regular follow-up calls, and motivational support will be provided to encourage adherence. Transportation stipends may be considered if budget permits.

Another potential difficulty is variability in dietary practices, which could confound metabolic outcomes. Although dietary practice is statistically controlled as a covariate, additional efforts will be made to monitor and reinforce consistent dietary behavior through brief nutritional guidance and regular check-ins.

Limited funding may restrict access to advanced diagnostic tools or biochemical assays. To mitigate this, cost-effective alternatives will be used where possible, and partnerships with local health institutions will be explored to access shared resources.

Data completeness and quality may also be affected by missing or inconsistent entries. This will be managed through rigorous data monitoring, double-entry verification, and early identification of discrepancies. Training sessions for data collectors and exercise supervisors will be conducted to ensure protocol fidelity.

These proactive measures aim to safeguard the integrity of the study and ensure successful delivery of outcomes within the stipulated timeframe and budget.

**Project management**

Successful implementation of this study relies on a multidisciplinary team with clearly defined roles and responsibilities to ensure scientific rigor, ethical compliance, and timely execution.

- **Principal Investigator (PI):** Leads the project, oversees protocol development, ethical approvals, and overall coordination. Responsible for supervising all study phases, ensuring adherence to timelines, and guiding data interpretation and dissemination.
- **Co-Investigators:** Support the PI in refining methodology, supervising data collection, and contributing to statistical analysis and manuscript preparation. They also assist in resolving implementation challenges and maintaining scientific integrity.
- **Exercise Physiologist:** Designs and supervises the exercise intervention, ensuring safety, correct sequencing, and protocol fidelity. Responsible for training exercise supervisors and monitoring participant progress.
- **Clinical Laboratory Professionals:** Conduct biochemical analyses. They ensure sample integrity, accurate processing, and timely reporting of results. Their role is critical for maintaining laboratory quality control and compliance with biosafety standards.
- **Data Manager:** Oversees data coding, entry, and verification. Ensures secure storage, monitors data quality, and prepares datasets for statistical analysis. Coordinates with the statistician to resolve discrepancies and manage missing data.
- **Statistician:** Develops and executes the statistical analysis plan, including RM ANCOVA modeling, power calculations, and imputation procedures. Supports interpretation of results and contributes to publication.

**Ethics**

Before deciding to participate, participants will be given a thorough explanation of all procedures, risks, and protocols, and will sign an informed consent form. Clearly outlining the steps the study team would take to protect patient privacy and confidentiality, including removing patient names and data. Health professionals should provide special consideration to the requirements and safety of diabetic patients during the study, as they are a vulnerable population. Furthermore, consulting the Research Ethical Review Board Sport Academy IRB in Debre Markos University ensures that an independent review board will assess the research procedures for ethical compliance. Finally, adhering to the ethical requirements of the Helsinki Declaration's 2000 revision guarantees the study prioritizes participant well-being and respects their rights.

To address the ethical concern of withholding exercise from the control group during the intervention period, the researcher will provide a tailored and supervised exercise program for these participants after the study is completed. This program will be designed to meet their specific needs and promote improvements in cardiovascular and metabolic health.

**Informed consent forms**

**Consent Form: (English Version)**

**Introduction:**

You are invited to participate in a research study investigating the effects of concurrent training order on cardiovascular function and biochemical markers in diabetic type II patients. This study aims to understand how different exercise sequences within concurrent training programs might impact your body's response on cardiovascular function and biochemical biomarkers.

**Procedures:**

- If you agree to participate, you will be asked to complete a medical screening to ensure your safety for the exercise program.
- You will be divided into groups and assigned a specific exercise sequence for concurrent training. This training will combine different types of exercise, like aerobic and resistance training for 12 weeks.
- Throughout the study, researchers will gather cardiovascular data (e.g., heart rate, blood pressure, and maximum oxygen uptake), biochemical biomarkers from blood samples (e.g., blood sugar, insulin levels, and lipid profiles), as well as anthropometric measurements (e.g., body weight, height, and body circumference) at two points: before and after the training program, to monitor participants' health and their response to the intervention.
- The entire study protocol will be explained by the researchers in detail, and you will have ample opportunity to ask questions before deciding to participate.
- If you are placed in the control group, which requires maintaining your usual physical activity routine throughout the intervention period, we understand the concern about potentially missing out on the benefits of exercise. To address this, a personalized exercise program will be offered to you after the study concludes. This program will be designed to support your cardiovascular and metabolic health. Details about this opportunity will be shared with you during the recruitment process.

**Confidentiality:**

All your personal information and medical data will remain confidential. A coding system will be used to de-identify your data during analysis and reporting to protect your privacy. Additionally, any blood samples collected for this study will only be used for the purposes of this research. Once the analysis is complete, these samples will be disposed of to address confidentiality concerns.

**Potential Risks and Benefits**

**Risks:**

- Exercise-related injuries (muscle soreness, strains)
- Potential discomfort during or following exercise sessions and assessments

**Benefits:**

- Improved overall fitness and metabolic health
- Increased knowledge about the effects of exercise order on concurrent training
- Free health assessments
- Contribution to scientific research

**Voluntary Participation:**

Your participation in this study is entirely voluntary. You have the right to withdraw from the study at any point without any penalty.

**Contact Information:**

If you have any questions about the research or your participation, please do not hesitate to contact the lead researcher,

| Name | Friew Amare |
| --- | --- |
| Email | firewa6070@gmail.com / friew_amare@dmu.edu.et |
| Phone No | +251913912776 |

**Note: *By signing this consent form, you acknowledge that you have read and understood the information provided above.*** ***If you decide to participate, you will be asked to sign this form.***

***Name of participant: __________________***

***Date: __________________***

***Signature: ____________***

***Name of Researcher: _________________***

***Date:__________________***

***Signature: ___________***

**Consent Form: (Amharic Version)**

**መግቢያ:**

ይህ ጥናት የኮንኮረንት አካላዊ ስልጠና በውስጡ የያዛቸዉ የስዉነት እንቅሰቃሴ የስልጠና ቅደም ተከተል ማለትም ኤሮቢከ እና ሪዚስታንስ የልብ እና የደም ዝውውር ተለዋዋጮች እንዲሁም የባዮኬሚስትሪ ምልክቶችን ላይ የሚያመጡትን ለዉጥ ለማጥናት የተዘጋጀ ነዉ። እርስዎም ይዚህ ጥናት ተሳታፊ እንዲሁኑ ትምርጠዋል።

**የጥናቱ ሂደት:**

- በጥናቱ ውስጥ ለመሳተፍ ፍቃደኛ ከሆኑ ድረስ ለስዉነት እንቅሰቃሴ ስልጠና ብቁ ስለመሆንዎ በጤና ምርምራ ልየታ ይደረግልዎታል።
- ተሳታፊዎቹ የተለያዩ የስልጠና ቅደም ተከተሎች እንዲሁም ክሁለቱ ዉጭ ልዩ ክትትል የሚደረግለት ከአካላዊ እንቅስቃሴ ነጻ (አካላዊ እንቅስቃሴ የማይሰሩ) ቡድኖች ውስጥ ሊመደቡ ይችላሉ።
- በጥናቱ ውስጥ የልብ የደም ዝዉውር ብቃት ምልክቶች (እንደ የልብ ምት ፣ የደም ግፊት እና ከፍተኛ የኦክሲጅን ጥልቅ ልቀት) ፣ የደም ምልክቶች (እንደ ደም ስኳር፣ ኢንሱሊን ሙግደት እና የሊፒድ መጠን) ፣ እንዲሁም የሰውነት መለኪያዎች (እንደ ክብደት፣ ቁመት እና የሰውነት ስብ ስሪት) በስልጠናዉ የመጀመሪያ እና የመጨረሻ ጊዜያት ውስጥ ከዕርስዎ ይሰበሰባል።
- የጥናቱ ሙሉ ሂደት በምርምር ቡድኑ ዝርዝር ማብራሪያ ይሰጣችኋል። ይህን ሲያደርጉ ማንኛውም ጥያቄ ለማድረግ ዕድል ይኖራችኋል።
- ልዩ ቁጥጥር በሚደረግበት ቡድን ውስጥ ማለትም (አካላዊ እንቅስቃሴ ከማይሰራዉ ቡድን) የተመደቡ ተሳታፊዎች መረመሩ እስከ ሚጥናቀቅ ድረስ መደበኛ የተለመደዉን እንቅስቃሴ ማስቀጠለ ይጠበቅባችዋል። ይህ ቡድን የምርምር ጊዜዉ እንዳልቀ ያልሰሩበትን ጊዜ ሊያካክስ በሚችል መንገድ የስልጠና ፕሮግራም የሚዘጋጂላቸዉ ይሆናል። ይህ ዕድል በምርምሩ ውስጥ ለተሳታፊንት በሚመለመሉበት ጊዜ በሰፊዉ ይብራራልዎታል።

**ሚስጢራዊነት፡**

ሁሉም የግል መረጃዎት እና የሕክምና መረጃዎች በሚስጥር ይያዛሉ። ምርምር ሲካሄድ የተሳተፉ ሰዎች መረጃ ማንነት በማይታወቅበት መንገድ መሆኑን ለማረጋገጥ ይጠቀማል። የደም ናሙናዎቹ ለምርምር ብቻ በመጠቀም ምርምሩ ከተጠናቀቀ በኋላ እንዲወግዱ ይደረጋል።

**የምርምር መሳተፍ ያለዉ ስጋትና ጥቅሞች:**

**ስጋቶች፡**

- በስልጠና ወቅት ሊክሰቱ የሚችሉ አካላዊ ጉዳቶች
- በስልጠና ጊዜም ይሁን በኋላ ላጭር ጊዜ የሚቆይ ምቾት የሌለዉ ሁኔታ መሰማት

**ጥቅሞች:**

- የአካል ብቃት እና ሁሉን እቅፍ የጤና ሁኔታን ማሻሻል
- የስልጠና ቅደም ተከተል በልብ እና በደም ምልክቶች ላይ ያለው ተፅእኖ ላይ ግንዛቤ እንዲኖራቸዉ ያደርጋል
- የነጻ የጤና ምርመራ
- ለሳይንስ ምርምር የራሰን አስተዋዖ እነደማድረግ ይቆጥራል

**የተሳታፊነት በጎ ፈቃድ:**

በዚህ ጥናት የሚሳተፉተ ያለምንም **አስገዳጂነት** በፍጹም ፍቃኝነት ነው። እንዲሁም ክምርምሩ በማንኛውም ጊዜ መዉጣት ውይም ማቋርጥ ይችላሉ።

**የምርምር አዘጋጅ:**

ስም: ፍሬው አማረ
ኢሜይል: [firewa6070@gmail.com](mailto:firewa6070@gmail.com) / [friew_amare@dmu.edu.et](mailto:friew_amare@dmu.edu.et)
ስልክ ቁጥር: +251913912776

የተሳታፊው ስም: _______________
ቀን: _______________
ፊርማ: _____________

የምርምር ተመራማሪ ስም: _______________
ቀን: _______________
ፊርማ: _____________

**Research protocol: part 2**

**Budget**

| Category | Details | Cost (ETB) |
| --- | --- | --- |
| Laboratory and Testing Costs |  |  |
| Biochemical Biomarker Tests | Blood sample testing (pre- and post-study) (1800.00*39*2) | 140,400.00 |
| Subtotal | | **140,400.00** |
| 3. Research Team |  |  |
| Research Assistants | 2 assistants for data collection (2*10,000.00) | 20,000.00 |
| Medical Professional | Fees for healthcare professionals to monitor participants during training (10,000*3 months) | 30,000.00 |
| Subtotal | | **50,000.00** |
| 4. Training Program |  |  |
| Payment for the trainer | 9000* 2 trainers * 3 months | 54,000.00 |
| Subtotal | | **54,000.00** |
| 5. Miscellaneous |  |  |
| Administrative Costs | Stationery, communication, and miscellaneous expenses | 4,200.00 |
|  | **Subtotal** | **4,200.00** |
|  | **Total** | **302,600.00** |

***Note****: All monetary values are shown in the local currency of Ethiopia.*

**Other support for the project**

This research project is fully funded by Addis Ababa University through its internal research support program. The funding is designated to cover core components of the study, including participant recruitment, laboratory testing, exercise supervision, data management, and follow-up procedures.

No external funding has been received or requested from other organizations. Importantly, the current budget does not include support for dissemination activities such as publication fees, conference presentations, or community outreach. The research team will seek alternative avenues for sharing findings once the study is complete, including institutional platforms and open-access options where feasible.

This funding reflects Addis Ababa University’s commitment to advancing locally relevant research in chronic disease prevention and non-pharmacological interventions.

**Curriculum Vitae of Investigators**

**Curriculum vitae of PI**

| 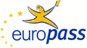 |  |
| --- | --- |
| **Europass Curriculum Vitae** | 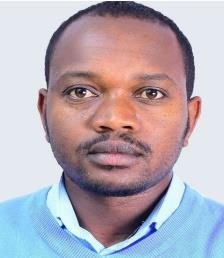 |
| First name and Surname | **Friew Amare Mengistu** |
| Telephone | +251 913912776 |
| phone | +251 587 716070 |
| E-mail | [firewa6070@gmail.com](mailto:firewa6070@gmail.com) |
| Nationality | Ethiopia |
| Date of birth | 1 April, 1990 |
| Gender | Male |

| **Publications** | - **Amare, F., Kiflu, A. & Taddese, A. Effects of concurrent continuous aerobic and short rest resistance exercise training on metabolic biomarkers in type 2 diabetes patients: a systematic review and meta-analysis. *Diabetol Metab Syndr* 17, 290 (2025).** [**https://doi.org/10.1186/s13098-025-01838-x**](https://doi.org/10.1186/s13098-025-01838-x) - **Amare, F., Alemu, Y., Enichalew, M. *et al.* Effects of aerobic, resistance, and combined exercise training on body fat and glucolipid metabolism in inactive middle-aged adults with overweight or obesity: a randomized trial. *BMC Sports Sci Med Rehabil* 16, 189 (2024).** [**https://doi.org/10.1186/s13102-024-**](https://doi.org/10.1186/s13102-024-00982-7)   [**00982-7**](https://doi.org/10.1186/s13102-024-00982-7)   - **Mengistu FA, Lake YA, Andualem ME, Miherete YD, Zewdie SA. Impact of aerobic, resistance, and combined training on cardiometabolic health-related indicators in inactive middle-aged men with excess body weight and obesity. Front Physiol. 2025 Feb 25;16:1519180. doi:** [**https://doi.org/10.3389/fphys.2025.151918**](https://doi.org/10.3389/fphys.2025.151918) - **Effect of Soy Protein Supplements on Handgrip Strength and Anaerobic Power Ability of Handball Players, International Research Journal of Modernization in Engineering Technology and Science(IRJMETS), Volume: 02/Issue:06/June-2020** - **Effects of ginger supplementation and aerobic training on body composition in obese women” in**   **International Research Journal Of Modernization In Engineering Technology And Science (IRJMETS), Volume 4, Issue 07, July 2022** |
| --- | --- |

**Curriculum vitae of Co-author 1**

| Contact Info. | Descriptions |
| --- | --- |
| Name | Alemmebrat Kiflu Adane (Associate Professor, PhD) |
| Email | [alemmebrat.kiflu@yahoo.com](mailto:alemmebrat.kiflu@yahoo.com)  [alemmebrat.kiflu@gmail.com](mailto:alemmebrat.kiflu@gmail.com) [alemmebrat.kiflu@aau.edu.et](mailto:alemmebrat.kiflu@aau.edu.et) |
| Institution | Ethiopia, Addis Ababa University, CNSCs, Departments of Sport Science |
| Mobile No. | +251- 09 39 25 36 50 |

| **NO** | **Degree** | **University Attend In:** | **Year G.c.** | **Specialization** |
| --- | --- | --- | --- | --- |
| 1 | Ph.D. | Andhra University India | August 23, 2010  -  August 25, 2013 | EXERCISE PHYSIOLOGY |
| 2 | MSc | Punjabi University, Patiala, India | June10, 2004  -  May 23, 2006 | EXERCISE PHYSIOLOGY |
| 3 | BSc | Kotebe College of Teachers Education Ethiopia | August 01, 1995  -  May 07,1999 | Health and Physical Education) |
| publications | | 1. **The Effect of Altitude Training on Physiological Variables of Endurance Athletes in Ethiopia,** [Physical Rehabilitation and Recreational Health Technologies](https://phrir.com/journal/index) 2. **Nordic Hamstring Curls are a Remedy for Hamstring Muscle Injury: A Narrative Review,**  International Journal of Human Movement and Sports Sciences, DOI: 10.13189/saj.2024.120411 3. **Effects of Altitude Training on Ethiopian Endurance Athletes Recovery Heart Rate and Haematological Variables.** Pedagogy of Physical Culture and Sports 4. **Relationship between anthropometric characteristics and team Performance in Ethiopian men volleyball premier league clubs,** The International Journal of Interdisciplinary Organizational Studies ISSN: 2324-7649 (Print), ISSN: 2324-7657 (Online) 5. **The Relationship between Preferred Leadership Style and Motivation among Ethiopia Female Volleyball Primer League Players, *Journal for Re Attach Therapy and Developmental Diversities,*** Online - 2589-7799 6. **Anthropometric Characteristics of Volleyball Players With Respect to Playing Positions: In Ethiopian Female Premier League**. Central European Journal of Sport Sciences and Medicine, ISSN (print): 2300-9705 \| ISSN (online): 2353-280 7. **Supervised Community Based Outdoor Group Exercise: As Prevention and Treatment of Arterial Blood Pressure and Body Weight,** NVEO – Natural Volatiles & Essential Oils. 8. **Effect of Brisk Walking and Ethiopian Great Lent on Body Weight and Lipid Profile: In People with Moderately Overweight**, International Journal of Multidisciplinary Educational Research. (ISSN: 2277-7881). Volume 8, Issue 4(3), Year 2019 9. **The Effect of 12-Weeks High Intensity Intermittent Exercise (HIIE) on Aerobic Capacity and Body Composition among Health Young Adults,** *International Journal of Multidisciplinary Educational Research (ISSN: 2277-7881).* | | |

| 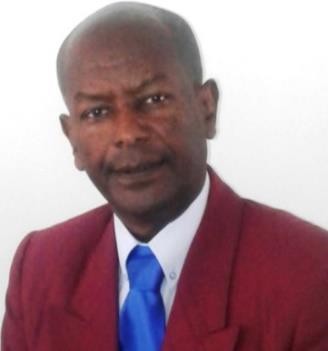 | Bio data  Name: Dr. Aschenaki Taddese Associate Professor, AAU Sex: Male  Marital status: Married Nationality: Ethiopian  Address:  Dr. Aschenaki Taddese Addis Ababa University, Ethiopia  Tel. 00251911 101661 or 00251983289446  Email: [aschenakitaddese1@gmail.com](mailto:aschenakitaddese1@gmail.com) |
| --- | --- |

**Curriculum vitae of Co-author 1**

Education : PhD in physical education, Osmania University Hyderabad, India. (2013)

1. **Publications**

- Chalachew Lemecha and Aschenaki Taddese Effects of plyometric and strength trainings on selected physical fitness variables in Ethiopia youth sport academy female soccer players *Ethiopian Journal of Science* SINET: Ethiop. J. Sci., 44(2): 234–241, 2021 ISSN: 0379–2897 College of Natural and Computational Sciences, Addis Ababa University, 2021 eISSN: 2520–7997
- Nigatu worku and Aschenaki Taddese The impact of hill training on middle and long distance athletes: with specific reference to Oromia water works athletics club, Ethiopia International Journal of Scientific and Research Publications, Volume 7, Issue 11, November 2017 287 ISSN 2250-3153 [www.ijsrp.org](http://www.ijsrp.org/)
- Addisu Firdu and Aschenaki Taddese Practices and challenges of implementing psychological skill training in Ethiopian youth sport academy and athlete Tirunesh Dibaba sports training center International Journal of Fitness, Health, Physical Education & Iron Games ISSN 2349 – 722X, Vol: 8, No: 1, Jan 2021 to June 2021
  - Aschenaki Taddese (2017) The effect of altitude on middle and long distance runners: International journal of health, physical education and computer science in sports. Volume

- 25, No. 1. January 2017 to March 2017

- - Aynalem Muchalo and Aschenaki Taddese (2016) Assessment of talent identification players’ success rate: With specific reference to Addis Ababa basketball project. International journal of health, physical education and computer science in sports Volume 23, No. 1 July 2016 - September 2016
  - Abdulaazziz Hassen and Aschenaki Taddese (2016) The effect of circuit training on some selected physical fitness components: With specific reference to Dessie town basketball project players, Ethiopia. International journal of health, physical education and computer science in sports Volume 22, No. 1 April 2016 to June 2016
  - Aschenaki Taddese (2015) Nature or nurture? The secret behind the distance king: An Ethiopian three gold and one siliver Olympic medalist, Kenenisa Bekele. International journal of health, physical education and computer science in sports Volume - 19 No. 1. July 15 – September 2015
